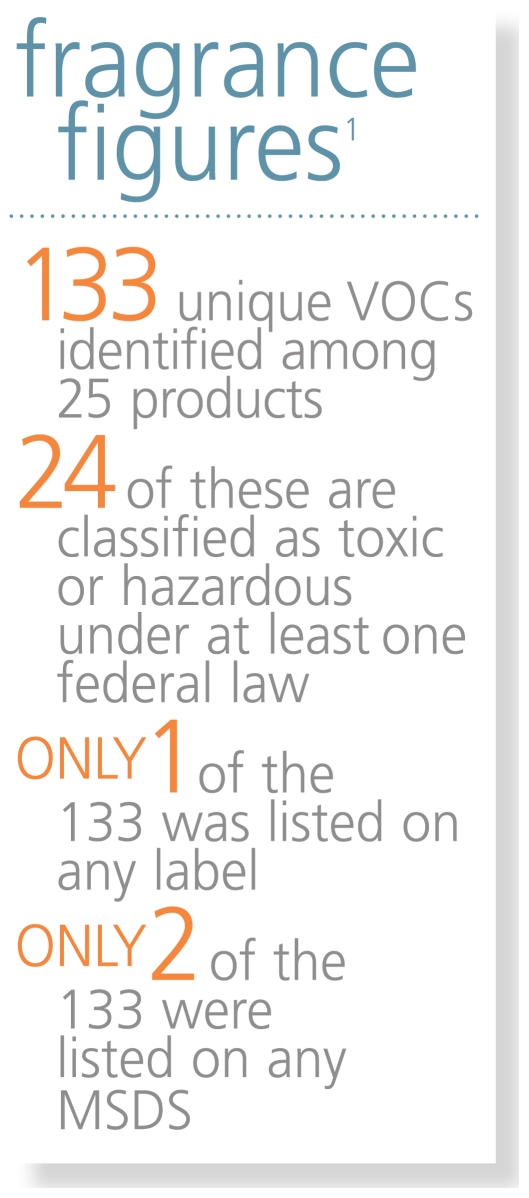# INDOOR AIR QUALITY: Scented Products Emit a Bouquet of VOCs

**DOI:** 10.1289/ehp.119-a16

**Published:** 2011-01

**Authors:** Carol Potera

**Affiliations:** **Carol Potera**, based in Montana, has written for *EHP* since 1996. She also writes for *Microbe*, *Genetic Engineering News*, and the *American Journal of Nursing*

A survey of selected scented consumer goods showed the products emitted more than 100 volatile organic compounds (VOCs), including some that are classified as toxic or hazardous by federal laws.^1^ Even products advertised as “green,” “natural,” or “organic” emitted as many hazardous chemicals as standard ones.

Anne Steinemann, a professor of civil and environmental engineering and public affairs at the University of Washington, Seattle, and colleagues used gas chromatography–mass spectrometry to analyze VOCs given off by the products. They tested 25 air fresheners, laundry detergents, fabric softeners, dryer sheets, disinfectants, dish detergents, all-purpose cleaners, soaps, hand sanitizers, lotions, deodorants, and shampoos. Many of the products tested are top sellers in their category.

A single fragrance in a product can contain a mixture of hundreds of chemicals, some of which (e.g., limonene, a citrus scent) react with ozone in ambient air to form dangerous secondary pollutants, including formaldehyde.^2^ The researchers detected 133 different VOCs. Most commonly detected were limonene, α- and β-pinene (pine scents), and ethanol and acetone (often used as carriers for fragrance chemicals).^1^

Steinemann and colleagues found the average number of VOCs emitted was 17.^1^ Each product emitted 1–8 toxic or hazardous chemicals, and close to half (44%) generated at least 1 of 24 carcinogenic hazardous air pollutants, such as acetaldehyde, 1,4-dioxane, formaldehyde, or methylene chloride.^1^ These hazardous air pollutants have no safe exposure level, according to the U.S. Environmental Protection Agency.^3^ Of the 133 VOCs detected, only ethanol was listed on any label (for 2 products), and only ethanol and 2-butoxyethanol were listed on any Material Safety Data Sheet (for 5 products and 1 product, respectively).^1^

The Consumer Product Safety Commission, which regulates cleaning supplies, air fresheners, and laundry products, currently does not require manufacturers to disclose any ingredients on the label, including fragrances in these products.^4^ The same is true for fragrances in personal care items, which are overseen by the Food and Drug Administration.^5^ The Household Product Labeling Act, currently under review in the U.S. Senate, would require manufacturers to label consumer products with all ingredients, including fragrance mixtures.^6^ “Disclosing all ingredients could be a first step to understanding potential toxicity and health effects,” says Steinemann.

Although the authors did not seek to assess whether use of any of the products studied would be associated with any risk,^1^ Steinemann says she receives hundreds of letters, phone calls, and e-mails from people who report a variety of respiratory, dermatological, and neurological problems they attribute to scented products: “Children have seizures after exposure to dryer sheets, and adults pass out around air fresheners,” she says.^7^ Steinemann and colleague Stanley M. Caress have written elsewhere that 19% of respondents across two U.S. telephone surveys reported health problems they attributed to air fresheners, and nearly 11% reported irritation they attributed to scented laundry products vented outdoors.^8^

“It’s important to take people’s complaints seriously,” says Steinemann, because “these human experiences are helping to inform science.” One of her next projects will focus on biomarkers of exposure and effect to better understand how fragranced products may cause a range of adverse health effects. “The ultimate goal is to improve public health,” Steinemann says. For now, she recommends cleaning with basic supplies like vinegar and baking soda.

Steinemann’s study “strongly suggests that we need to find unscented alternatives for cleaning our homes, laundry, and ourselves,” says Claudia Miller, an allergist and immunologist at the University of Texas Health Science Center at San Antonio. An expert in chemical sensitivity, or toxicant-induced loss of tolerance, Miller created the Quick Environmental Exposure and Sensitivity Inventory,^9^ a screening tool for chemical intolerance. According to Miller, products intended to keep homes smelling fresh can set people up for a lifetime of chemically induced illness, and repeated exposure to small amounts of household chemicals can trigger symptoms to previously tolerated chemicals.^10^ “The best smell is no smell,” Miller says.

## Figures and Tables

**Figure:**